# Mapping a Plasmodium transmission spatial suitability index in Solomon Islands: a malaria monitoring and control tool

**DOI:** 10.1186/s12936-018-2521-0

**Published:** 2018-10-22

**Authors:** Isabelle Jeanne, Lynda E. Chambers, Adna Kazazic, Tanya L. Russell, Albino Bobogare, Hugo Bugoro, Francis Otto, George Fafale, Amanda Amjadali

**Affiliations:** 1 Australian Bureau of Meteorology, Melbourne, VIC Australia; 2 Australian Institute of Tropical Health and Medicine, James Cook University, Cairns, QLD Australia; 3 Ministry of Health and Medical Services, National Vector Borne Disease Control Programme, Honiara, Solomon Islands; 4 Research Department, Solomon Islands National University, Honiara, Solomon Islands; 5 Pacific Science Solutions, Suva, Fiji

**Keywords:** Malaria, Solomon Islands, *Anopheles farauti*, Risk mapping, Suitability, Transmission, Land cover

## Abstract

**Background:**

Malaria remains a challenge in Solomon Islands, despite government efforts to implement a coordinated control programme. This programme resulted in a dramatic decrease in the number of cases and mortality however, malaria incidence remains high in the three most populated provinces. *Anopheles farauti* is the primary malaria vector and a better understanding of the spatial patterns parasite transmission is required in order to implement effective control measures. Previous entomological studies provide information on the ecological preferences of *An. farauti* but this information has never before been gathered and “translated” in useful tools as maps that provide information at both the national level and at the scale of villages, thus enabling local targeted control measures.

**Methods:**

A literature review and consultation with entomology experts were used to determine and select environmental preferences of *An. farauti*. Remote sensing images were processed to translate these preferences into geolocated information to allow them to be used as the basis for a Transmission Suitability Index (TSI). Validation was developed from independent previous entomological studies with georeferenced locations of *An. farauti*. Then, TSI was autoscaled to ten classes for mapping.

**Results:**

Key environmental preferences for the *An. farauti* were: distance to coastline, elevation, and availability of water sources. Based on these variables, a model was developed to provide a TSI. This TSI was developed using GIS and remote sensing image processing, resulting in maps and GIS raster layer for all the eight provinces and Honiara City at a 250 m spatial resolution. For a TSI ranging from 0 as not suitable to 13 as most suitable, all the previous collections of *An. farauti* had mean TSI value between 9 and 11 and were significantly higher than where the vector was searched for and absent. Resulting maps were provided after autoscaling the TSI into ten classes from 0 to 9 for visual clarity.

**Conclusions:**

The TSI model developed here provides useful predictions of likely malaria transmission larval sources based on the environmental preferences of the mosquito, *An. farauti*. These predictions can provide sufficient lead-time for agencies to target malaria prevention and control measures and can assist with effective deployment of limited resources. As the model is built on the known environmental preferences of *An. farauti*, the model should be completed and updated as soon as new information is available. Because the model did not include any other malaria transmission factors such as care availability, diagnostic time, treatment, prevention, and entomological parameters other than the ecological preferences neither, our suitability mapping represents the upper bound of transmission areas. The results of this study can now being used as the basis of a malaria monitoring system which has been jointly implemented by the Solomon Islands National Vector Borne Disease Control Programme, the Solomon Islands Meteorological Services and the Australian Bureau of Meteorology. The TSI model development method can be applied to other regions of the world where this mosquito occurs and could be adapted for other species.

## Background

In the Western Pacific Region, around 730 million people experience some risk of malaria, with over 30 million deemed as being at high risk [1]. The risk of malaria transmission is the highest in Papua New Guinea, the Solomon Islands and Vanuatu where more than 90% inhabitants are living in a high malaria transmission risk area, with the Solomon Islands and Papua New Guinea accounting for 86% of all reported deaths in the region in 2014. The transmission of malaria is both spatially and temporally variable, and understanding such variability is essential to improving control and elimination programmes.

Within the Solomon Islands the seasonal variability of malaria is well known but knowledge gaps remain. Current geographical categories of the Health Divisions used by the malaria control programme are too broad and have environmental heterogeneity that makes it difficult to effectively analyse the links between environment and malaria transmission and therefore to implement more finely targeted control measures. A single health division can contain both coastal and inland villages experiencing different risk profiles and requiring different approaches to reduce infection rates. As in many other malaria impacted regions of the world, there is need to improve systems used for high-quality surveillance, monitoring and evaluation, including the development of new tools to identify changes in disease burdens and risk levels [1, 2].

The Solomon Islands is a great case study for demonstrating the utility of high-resolution GIS techniques for improving the understanding of malaria transmission providing a baseline for improved malaria control efforts. In the Solomon Islands, the government implemented control program that included house spraying with dichlorodiphenyltrichloroethane (DDT), insecticide-treated bed nets and community awareness programmes [3, 4] was interrupted in the early 2000s due to ethnic violence. A subsequent large increase in the number of malaria cases led to a new government “National Malaria Strategic Vision, 2007–2016”, which aimed to reduce national malaria incidence by over 75%, including malaria elimination in the provinces of Temotu and Isabel (National Malaria Strategic Vision 2007–2016). This programme was largely successful, with a dramatic drop in infection rates. The annual parasite incidence is now below 100 cases per 1000 population in all provinces [1, 4], although there is considerable spatial variability in incidence, with incidence remaining relatively high in three of the more populated provinces, Guadalcanal, Malaita and Central Province [2]. High resolution geographical information system (GIS) mapping was implemented in the two malaria elimination provinces, Isabel and Temotu, to organize reactive treatment around each new case [5]. This included obtaining global positioning system (GPS) coordinates of households over several years, but did not include any predictive capacity or environmental transmission risk.

In order to further reduce the malaria burden in the Solomon Islands, tools are required that can more accurately map malaria transmission risk at a spatial resolution that allows for targeted and cost effective control programs at the scale of villages including all the high incidence areas. Identifying locations that are suitable breeding sites for the main malaria vector, *Anopheles farauti* is needed to manage control strategies over the country and in each province and for the management of local larval sources at the scale of villages. This is achieved via the following approach: (1) identify the ecological preferences of the primary mosquito vector for malaria in the Solomon Islands, *An. farauti*; (2) use of high-quality data to build GIS layers based on key variables related to these ecological preferences, as well as locations of human settlements; (3) develop monitoring tools in the form of maps and GIS layers, for users to identify likely regions of malaria transmission.

## Methods

### Study area

**Fig. 1:**
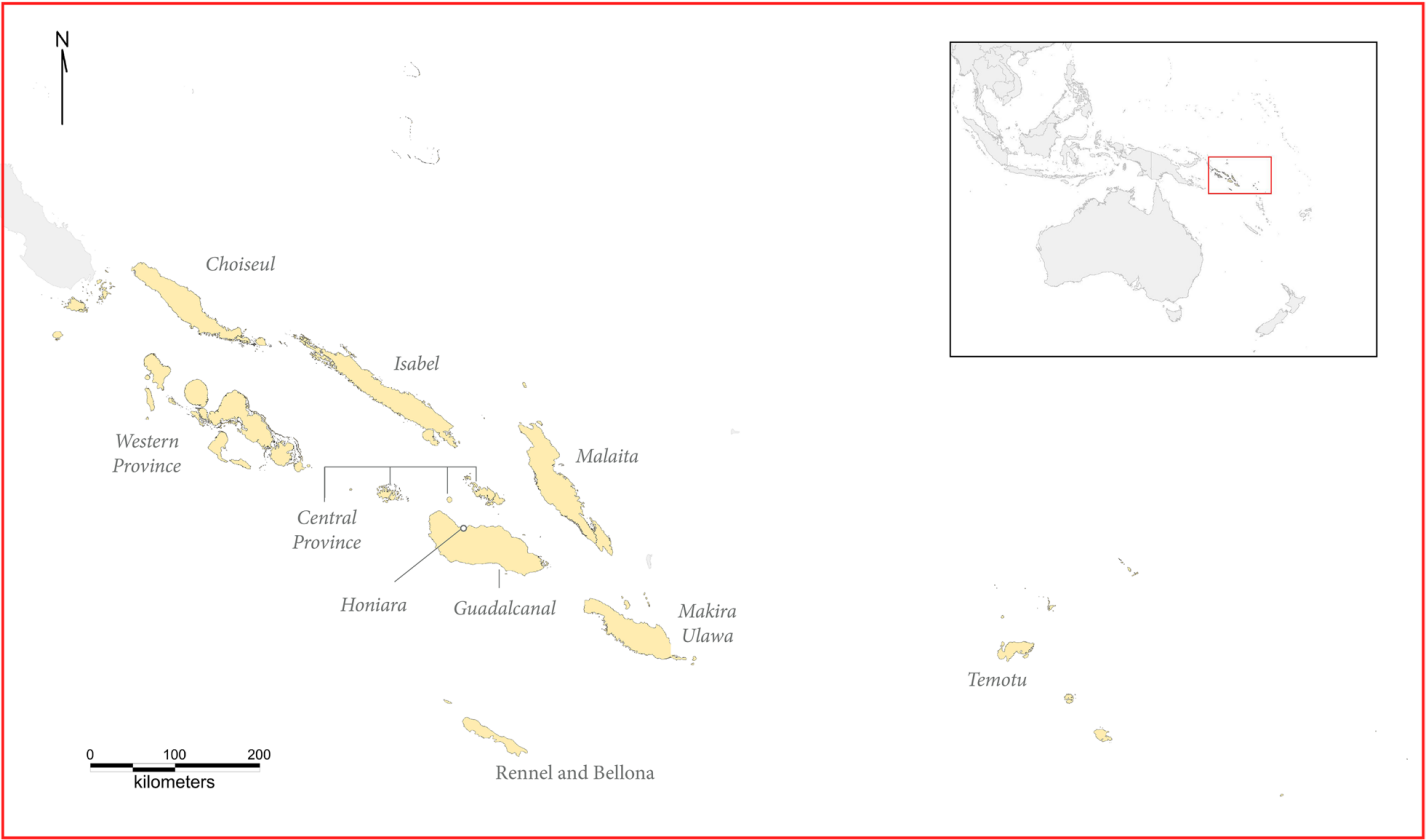
Solomon Islands’ provinces. In italic, the eight provinces and Honiara City where malaria cases occur

The Solomon Islands lie between 5° and 12°S and 152° to 170°E. Made up of over 900 islands in the Western Pacific, the Solomon Islands has ten administrative divisions. Malaria occurs in all but one of these, Rennel and Bellona Province (Fig. 1). The WHO lists the Solomon Islands as one of their malaria elimination programme countries as the National Vector Borne Disease Control Programme (NVBDCP) aims to eliminate malaria in two of the peripheral areas, Isabel and Temotu Provinces, and to decrease the high levels of malaria incidence in Guadalcanal Province [4].

The Solomon Islands has a tropical climate and the major islands have often steep thickly forested mountain ranges and deep narrow valleys [6]. Coastal areas consist of coral reefs, lagoons and mangrove swamps. Rainfall occurs throughout the year, with higher rainfalls between November and April. The majority of the population reside within 5 km from the coast on the coast, with the only substantial inland populations occurring in Guadalcanal and Malaita [7].

The largest island is Guadalcanal, which also has the highest rate of malaria risk. There has been a dramatic decrease in the number of confirmed malaria cases in the last 20 years due largely to increased awareness, prevention measures, and access to care [1, 4]. However, malaria remains an important health issue and further progress towards their aims of elimination and control will rely on having in place improved surveillance and monitoring systems.

### *Anopheles farauti* behaviour and ecological preferences

In order to build a malaria risk model it was important to identify environmental variables that indicate the ecological preferences of the main mosquito vector for the Solomon Islands, *An. farauti*. This was achieved by reviewing the literature and consulting experts. The literature review was primarily conducted using PubMed, supplemented through searches using Google Scholar and available archives (http://www.acq.osd.mil/eie/afpmb/content/literature-retrieval-system). The experts that were directly contacted were Dr. Nigel Beebe (University of Queensland) and Dr. Patricia Dale (Griffith University) at the beginning of the study and Professor Thomas Burkot (James Cook University) and Dr. Robert Cooper (Australian Army Malaria Institute) at the validation stage.

Proximity to water sources, including those around settlements, and distance from the coast influences the presence of *An. farauti*. This species is known to breed in a wide range of water sources, from fresh water, small containers, including coconut shells, to brackish pools [6]. However, this species is more common close to the coast, particularly in coastal swamps and low-lying riverine areas [6, 8], and is rarely seen beyond 5 km from the ocean. More recently, in Central and Western provinces, a large larval study showed that most common habitats of *An. farauti* larvae were coastal lagoons and swamps [9]. The most productive sites were few, fixed and findable making them amenable for larval source management.

The flight range of *An. farauti* is around 1–1.5 km [10]. Charlwood et al. captured *An. farauti* in two villages separated by 4 km and then released them either in their original village or the other one. Displaced mosquitoes were recaptured in both villages but non-displaced ones were not [11]. Elevation is thought to play an indirect role in the presence of *An. farauti* through its impact on temperature and humidity [12]. Temperature both affects the survival rates of the parasites during the stage inside the Anopheles as well as that of the mosquitoes themselves. The ideal temperature range for the parasite is 19–25 °C [13]. Soil temperature is also important to survival of the mosquitoes and this too is influenced by elevation, as well as by aspect. Temperature decreases with altitude at a rate of around 1 °C for every 100 m in elevation, though the relationship is weaker near the equator [14]. Within the Solomon Islands, temperatures and relative humidity tend to be relatively stable and within the ideal limits for both the parasite and the mosquito, however, on the larger islands such as Guadalcanal and Makira there is a rain shadow effect [14, 15]. In these locations greater rainfall is experienced on the windward side of the islands than on the more sheltered (by higher elevation areas) leeward side. However, aspect (in the geographical term of direction which the slope is facing) is not considered further in the model as it does not impact on the presence of the mosquito, but rather on *Anopheles* quantity and seasonal variation. *Anopheles farauti* has only once been found at more than 100 m altitude (in Vanuatu, at 335 m; [10]). However, *An. farauti* sensu lato species are isomorphic and at the time of Daggy’s study [10], before molecular-based techniques described in 1991 and 1995 [16, 17], *An. farauti* was indistinguishable from its siblings species, one of which has been collected at higher elevations. Hence, elevation may only be an indirect factor for the presence of *An. farauti*.

Based on the review of the literature and consulting entomological experts, a list of environmental parameters that are expected to impact on the biology or behaviour of *An. farauti* was generated. The initial list was further refined to ensure that the final parameters selected could be easily translated into a format that could be later used within an operational model to help control the spread of the mosquito. Where two possible sources were available for the same parameter, preference was given to that which had few data gaps and was more likely to be easily available into the future.

### Model development

The model developed for monitoring likely regions of malaria transmission was based on a spatial translation of what is known about *An. farauti* in the Solomon Islands. It is a statistical model based on published field information, and it is not considered to be a physiological model.

#### Scores

Once the environmental parameters were chosen (see *Results* for further details), values of these parameters were converted into scores. These scores consisted of ordered categories, the number and thresholds of which were determined using published results from field studies and a synthesis of knowledge from a review of the literature. To allow for standardization between variables, all scores were scaled to be within the range 0–5. The final scores for each parameter were further validated through consultation with entomological experts.

### Suitability model

A model for malaria transmission suitability was developed using available knowledge and parameter scores, i.e. using a partially theoretical structure combined with data. While it is acknowledged that the ecological preferences component may be incomplete, as there are some existing gaps in knowledge, this model allows for parameter adjustment and for the output of new spatial information based on the assembly of several parameters known to impact on *An. farauti* presence. The result is a spatial prediction model at much finer scales than previously available, i.e. at the scale of villages and the malaria control programmes.

The transmission suitability index, TSI, for malaria in the Solomon Islands was used is an additive model with weighed parameters of the form:

$$ TSI\, = \,w_{ 1} \,*\,p_{ 1} \, + \,w_{ 2} \,*\,p_{ 2} \, + \, \cdots \, + \,w_{k} \,*\,p_{k} $$

where *w*_*i*_ is the weight of the *ith* parameter, *p*_*i*_.

The formula and resulting preliminary suitability maps were presented to entomology experts for validation. The resulting TSI, which ranged from 0 to 13, was used for the validation and then was autoscaled into 10 classes, to 0–9 for the mapping.

### Data collection and preparation

#### Malaria cases data

The National Vector Borne Disease Control Programme (NVBDCP) is responsible for monitoring and control of malaria within the Solomon Islands. As part of this process they are responsible for maintaining a malaria database which contains monthly passive case detection data. They provided monthly malaria cases and Solomon Island GIS data on administration, health and environment.

Available monthly passive case detection data are aggregated at the provincial level from 1988 to 1998, but were also available at the sub-provincial level, from 1996 for Honiara City, Central Province and Isabel Province, from 1997 for Guadalcanal Province, from 1999 for Temotu Province and from 2000 for Makira-Ulawa and Western Provinces. In 2001, new divisions were introduced. However, data from 2000 and 2001 were incomplete and considered unreliable due to ethnic conflicts during this period. As a result, cases from 2001 to 2012 as reference were used.

The malaria data were checked for potential errors, including double, misspelt, misnamed or missing data. The cleaned data were then compared to a GIS database that included health centre locations and names. Data errors and location or name conflicts were corrected, where possible, and some remaining questions were checked during a workshop with the national and province malaria control managers in Honiara. This resulted in all health centres from the malaria cases database being georeferenced by health area and province for each of eight provinces and Honiara city. Finally, a buffer of 5 km was defined around each health centre and a specific GIS layer was allocated, because distances greater than 5 km have been shown to have negative impact on health [18].

#### Administrative and settlement data

Information was obtained on the administrative boundaries, health administrative points and areas and settlement locations. For the administrative boundaries this was through the database of global administrative areas (GADM; http://www.gadm.org/) which contained information on the country, province and wards (polygons). The health administrative data were obtained from Solomon Islands GIS, provided by the NVBDCP. Census data, obtained from the Solomon Islands National Statistics Office, 2009 Census of Population and Housing, through the PopGIS2 platform (Secretariat of the Pacific Community, Statistics for Development Division), was used to locate settlements. The Census indicating around 9000 settlements in the Solomon Islands. All data were checked for double, misspelt, misnamed or missing values. The administrative contours from GADM were then used to calculate the distance to the coastline, after validation by image process calculations. The final results were GIS layers for settlements, health centres, and settlement distance to a health centre, inside or outside a buffer of 5 km. Around 678 settlements were within 5 km of at least two health centres, 6580 within 5 km of one health centre and 2442 over 5 km from any health centre. This data could then be used in GIS queries and maps.

#### LANDSAT ETM + band 8

LandSat 7 ETM + band 8, sourced from the Global Land Cover Facility (http://www.landcover.org), was used to calculate higher resolution information on the distance to the coast. The ETM or enhanced thematic mapper Plus sensor has images with a 30 m spatial resolution over seven spectral bands. The eighth band has a 15 m resolution and was the only one used in this analysis. Landsat products could not be used for land use and land cover determination because of the small numbers of available images and the frequent cloud cover. One image was identified to have no cloud on the Guadalcanal coastline and was used as the GADM source for the Island/Province boundary. Further details on the images used are provided in Appendix A.

#### Elevation

Digital elevation data were obtained from the Shuttle Radar Topography Mission Version 4 (SRTM V4) Version 4 courtesy of the US Geological Survey (http://srtm.csi.cgiar.org) [19, 20]), using the C-band Wavelength (5.6 cm). These data were arranged in 1° by 1° tiles, resampled to a resolution of 3-arcs second, 90 m at the equator. The vertical datum used was the EGM96 (Earth Gravitation Model 1996), the vertical error in the digital elevation model being less than 16 m. Further details on the images used are provided in Appendix A.

#### Land cover

Land cover data were obtained from the European Space Agency and the Université Catholique de Louvain (http://due.esrin.esa.int). This Global Cover 2009 dataset [21] has a 250 m spatial resolution, based on the MERIS sensor on the ENVISAT satellite, and uses 22 land cover classes defined by the FAO Land Cover Classification System. The data were processed using a window on the Solomon Islands and then for each of the provinces. Provided in a Plate-carrée projection, a plane equi-rectangular projection, the data were georeferenced with resampling by nearest neighbour method (with IDRISI software), so as to be converted into the same projection, as the other layers. The total root mean square error was less than half the size of the cell (15/2 m) and then verified upon importing into GIS software. The data within the images were classified according to level of water coverage: i.e. no water, permanent water, and non-permanent water with potentially flooded areas. Further details on the images used are provided in Appendix A.

#### Software

R software version 3.4.3 [22] with the following packages ggplot2 [23], tidyverse [24] were used for statistical analysis and graphs. Images were processed in TerrSet 18.21 © 1987–2016 J. Ronald Eastman [25], products images were exported in geotif format, then imported into Mapinfo Pro 15.0 © 2015 Pitney Bowes Software Inc., where GIS layers were organized and GIS queries were dealt with.

## Results

The main results are separated according to the identified ecological preferences of the main mosquito vector, *An. farauti*, and settlement locations, leading to the development of maps and a GIS layer of the suitability index. Literature review and expert consultation identified the following environmental variables were identified to significantly contribute to the likely presence of *An. farauti*: distance from coast, elevation, presence of water bodies and proximity to settlements (as humans are a blood source).

### Distance to coastline

Prior to calculating the distance of settlements from the coastline, the distance of all pixels from the coastline was determined from LANDSAT—band 8 images, described in the “Methods” section. Three images were used, all from the 4th of April 2001, before the scan line corrector (SLC) failed in May 2003 resulting in data gaps. An additional image, from the 12th of January 2006, was used. This latter image retained the same radiometric and geometric corrections as the earlier images (https://landsat.usgs.gov). A mosaic, or merge, of the images was then used to create a single image covering the Solomon Islands.

Data from mosaic image covering the Guadalcanal Province was extracted and verified against the GADM dataset. This was achieved by creating a new image with the same parameters, spatial resolution and corner coordinates, and rasterizing the contour polygon. A mask was then created for the Guadalcanal Province, with values of 0 for sea and 1 for land pixels. An Edge filter, Laplacian edge enhancement 3 × 3, was used to ensure the province area had a minimum width of 2 pixels and a continuous boundary. This final image was created by overlaying the mask image to ensure that distance from the coast was only calculated using pixels inside the province boundary (contour). As a good match was obtained between the mosaic image and the GADM dataset, this contour extraction validation step was not required for the other provinces.

**Fig. 2:**
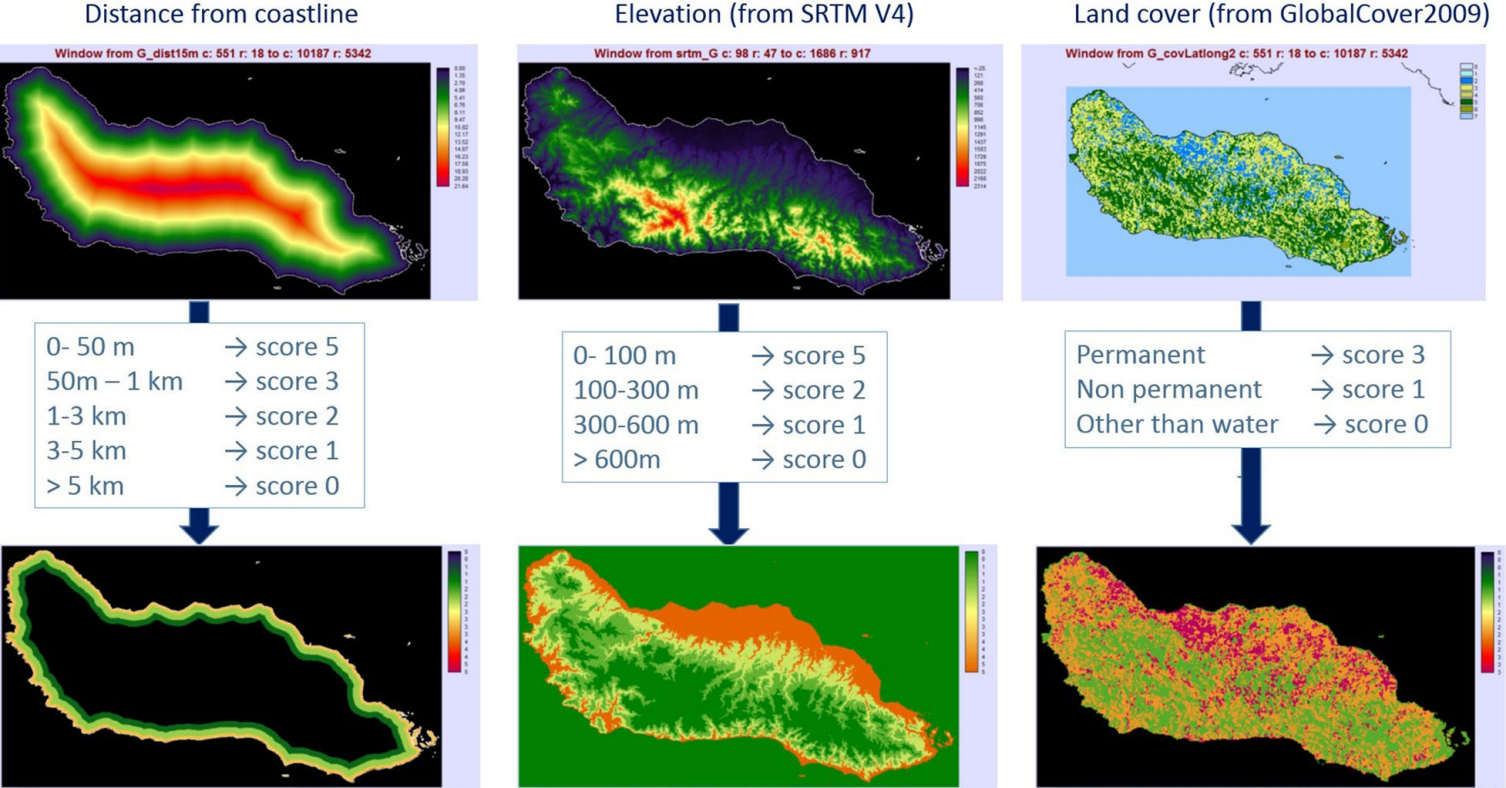
Distance to coastline—image processing. (1) From a mosaic of two LANDSAT images (15 meters spatial resolution) Guadalcanal main island is extracted; (2) A new image of the same spatial resolution is created with a rasterization of Guadalcanal’s surface area; (3) A filter is created to extract the contour of the island to be able to calculate the distance of each pixel of this image to the contour; (4) A multiplication of each pixel distance value by 0 for the sea pixels or by 1 for Guadalcanal island pixel to allow a better display of the distance to the coast for each pixel of the island

To translate these scores of the distance from the coast for each pixel (in metres), we used the IDRISI software (https://clarklabs.org/) and converted this from degrees to metres using the following procedure. The distance in degrees was multiplied by the length of a degree at Guadalcanal which equals 109.6251 km (= [2 * cos(latitude) * 6378]/360, where 6378 is the radius of the Earth in km) and then converted to metres. Calculation of the distance of settlements from the coast is described in “Model” section. The process obtaining the distance from coast data is described in “Model” section and shown in Fig. 2.

The need for a high spatial resolution for distance to the coastline supported by the study of Bugoro et al., where larvae were sampled at 50 m intervals along three streams, starting from the coast [26]. Larvae density followed a J-shaped curve, decreasing from 0 to 50 m to 500 m from the coast and monotonically decreasing until 3 km. Based on these results we determined non-linear scores for distance to coastline (see Table 1).

**Table 1:** Score assigned to factors

Score	Distance to coastline (m/km)	Elevation (m)	Landcover
5	0–50 m	0–100 m	–
4	–	–	–
3	50 m–1 km	–	Permanent water bodies
2	1–3 km	100–300 m	
1	3–5 km	300–600 m	Non-permanent water bodies, including irrigated cropland or flooded
0	> 5 km	> 600 m	No water, no flood, no irrigation

### Elevation

Elevation may be an indirect factor of *An. farauti* presence through its impact on localized temperature and humidity [12]. Temperature and humidity are also known to influence the survival of the internal parasites of the mosquito. Four SRTM Version 4 geotif images of digital elevation data were used as a mosaic to cover the Solomon Islands and a window was determined for each of the eight provinces and Honiara city. Pixels values that were below sea-level elevations reclassified as 0 m (using IDRISI software). A score classification was then applied (Table 1). The data were then exported in bmp format and the resulting raster images georeferenced in GIS software.

### Proximity to water bodies

The Global Cover 2009 dataset [21] was used to determine locations and classification of water bodies. As the image was in Plate-Carrée projection, it had to be georeferenced and resampled.

As the permanence of water (natural or irrigated) was the most important characteristic, these were reclassified, and assigned a score, as follows: Permanent water bodies, permanently flooded areas (score = 3) corresponding to Global Cover code 210, 170. Non-permanent (regularly flooded or irrigated) water bodies and waterlogged soil by fresh, brackish or saline water (score = 1): Global cover code 11, 14, 160, 180. No water, no flood, no irrigation (score = 0): Global Cover, all other codes (Table 1).

### Model

The model was developed in two main steps: creation of a Boolean raster image for settlement distance and then, reclassification and standardizing of the raster images for distance from the coastline, elevation and land cover.

#### Boolean raster image of settlement proximity

A Boolean raster image was created with distance from settlement as a variable. A value of 1 was attributed to each pixel that was within 1 km of any settlement, while a value of 0 was assigned to pixel greater than 1 km from a settlement.

#### Reclassification, weight and standardization

**Fig. 3:**
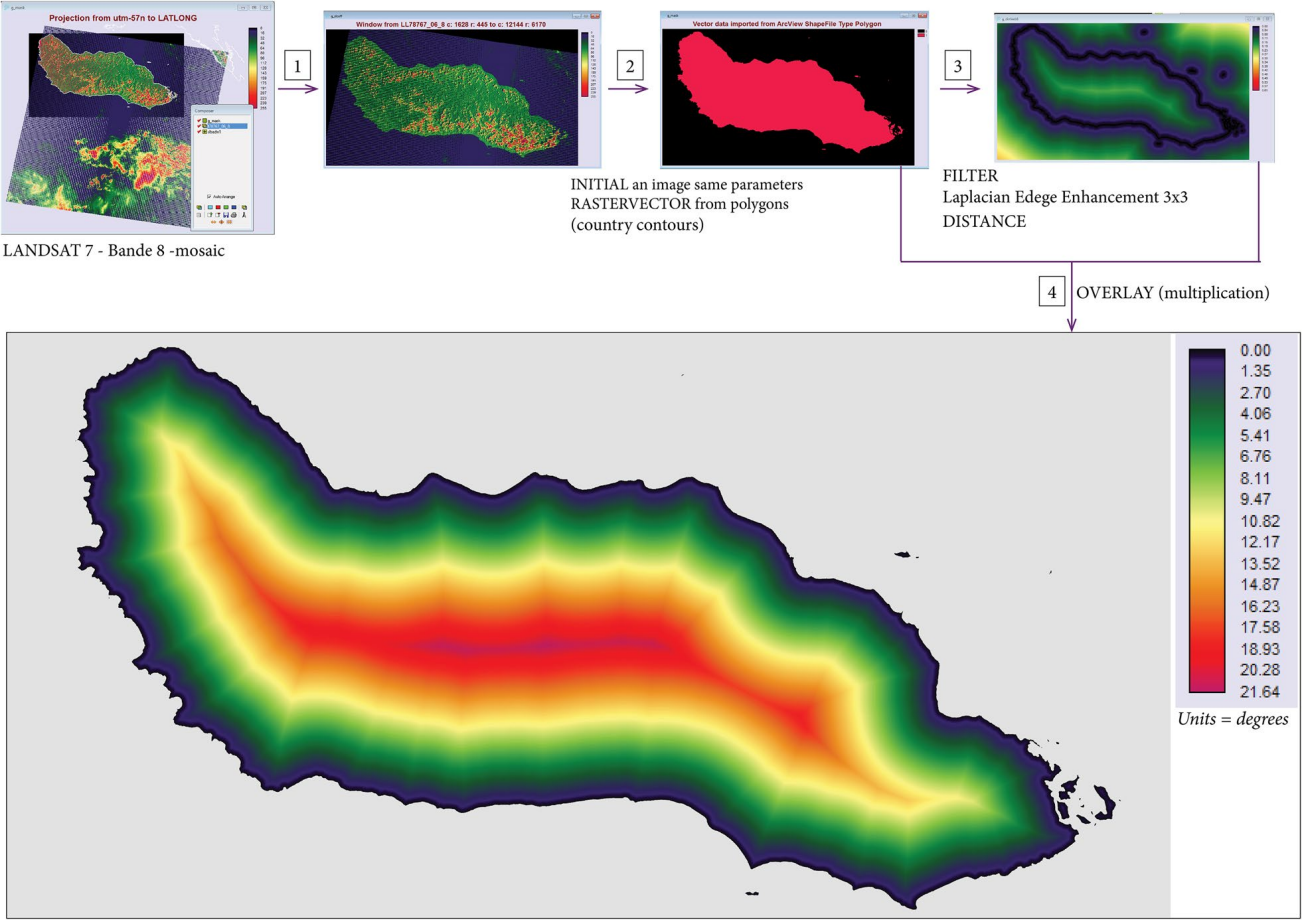
Scores. Distance to coastline, Elevation and Land cover scores used in the model. From literature and experts consultation, a score between 0 and 5 has been assigned to each factor (distance to coastline, elevation, presence of water)

A new integer value between 0 and 5 was assigned to each pixel of the three following raster images: distance from the coastline, elevation and land cover (Table 1 and Fig. 3).

### Model development

To develop the model of malaria transmission suitability, the final raster images for distance from coastline, elevation and land cover were selected and the score from each image for each pixel were added, using a logical OR operation. A logical AND operation was then used to multiply the settlement images with the resultant image. The final image had new pixels ranging in value from 0, if its geolocation was in the sea or inland and more than 1 km from any settlement, to the sum of the scores of distance from coastline, elevation and land cover (maximum value of 13) if not. The final model for transmission suitability index, as described above, is represented by the formula:

*TSI *=* w1* * (distance from coastline) + w2 * (elevation) + w3 * (land cover); for geolocations less than 1 km from a settlement; and 0 otherwise, where *w1*, *w2*, *w3* are the scores associated with distance to coastline, elevation and land cover, respectively (range 0–5).

**Fig. 4:**
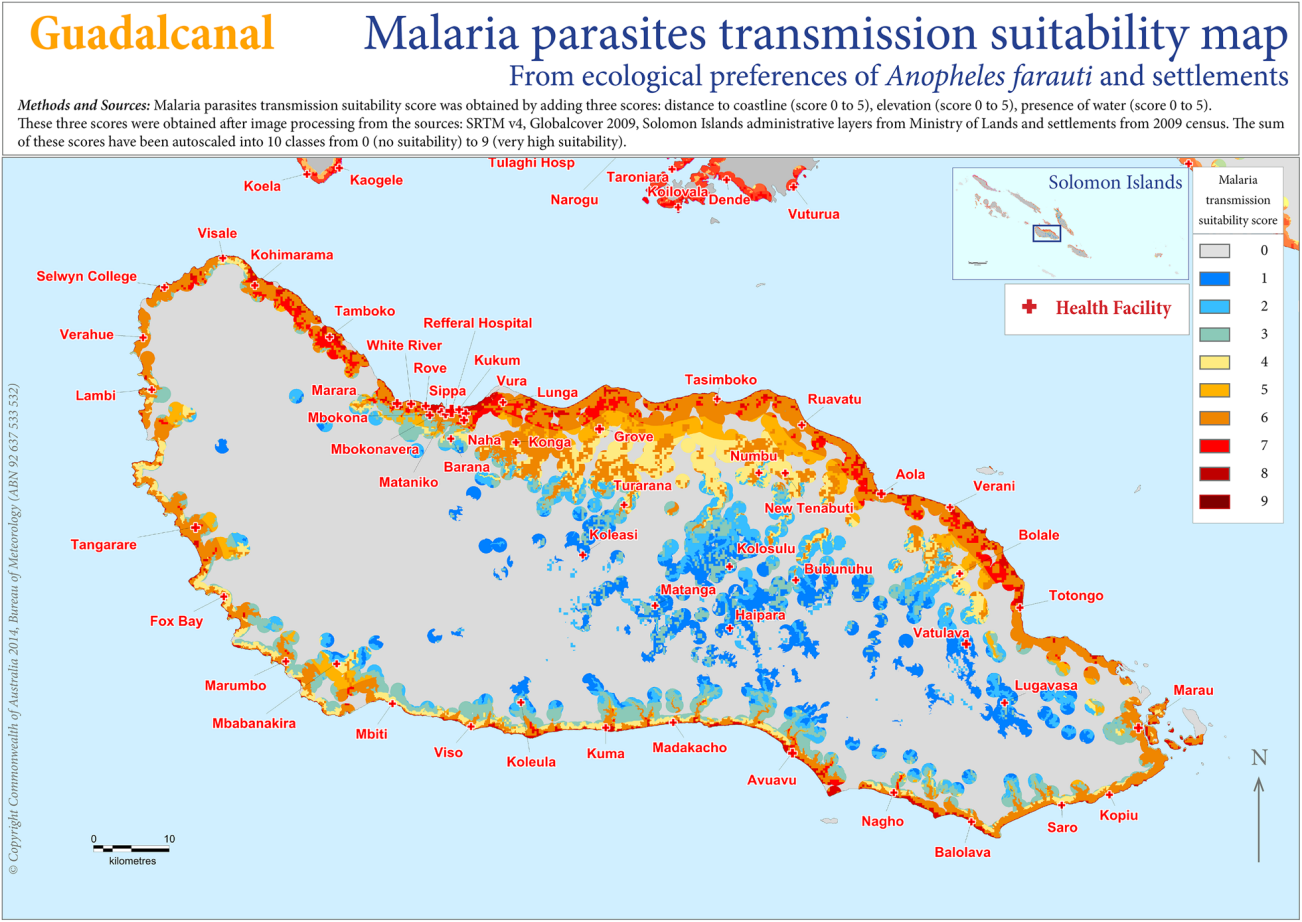
Plasmodium transmission spatial suitability and health centres map for Guadalcanal

**Fig. 5:**
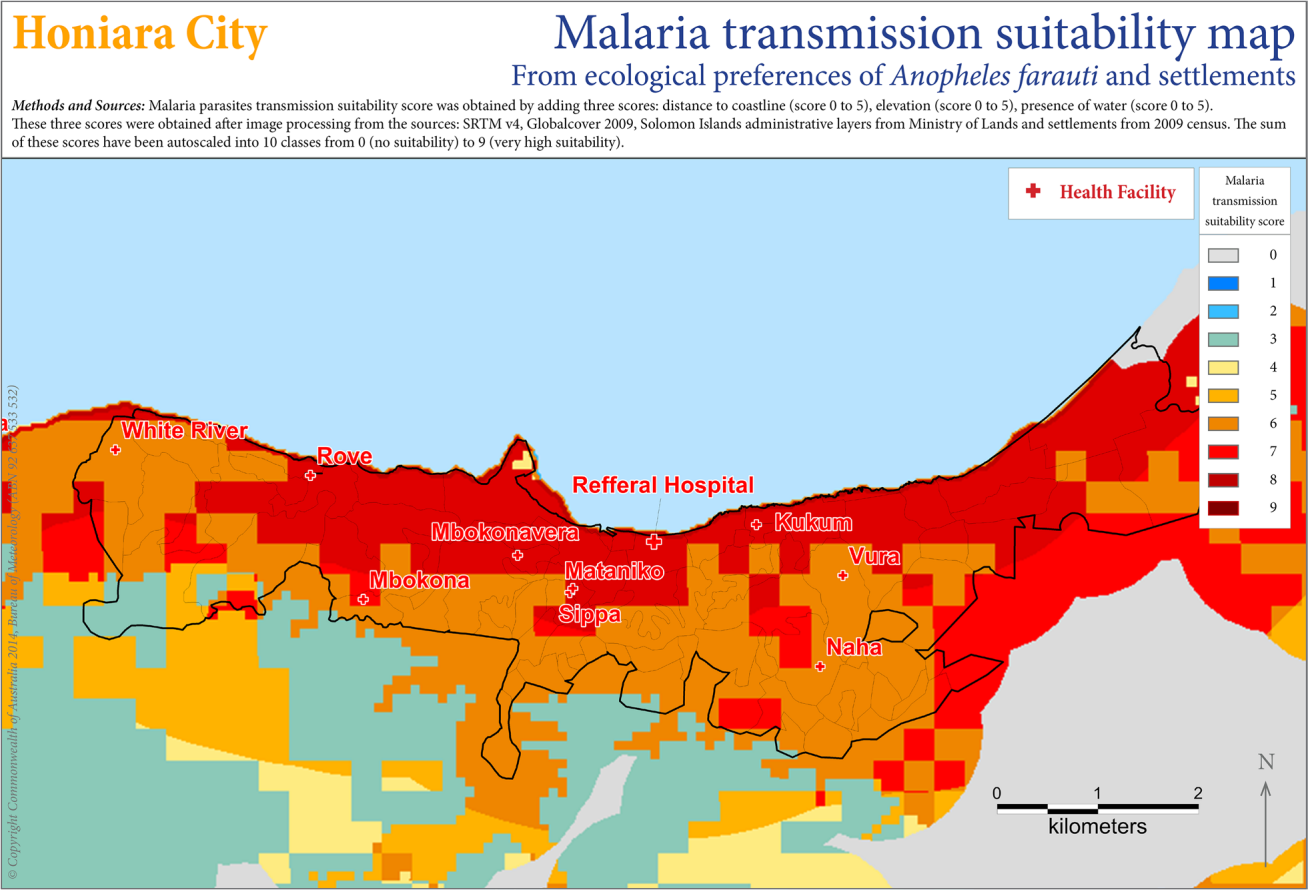
Plasmodium transmission spatial suitability and health centres map for Honiara City

See the suitability map for Guadalcanal in Fig. 4 and Honiara City in Fig. 5 and in Appendix B for details and for the other provinces.

### Model validation

Validation of a suitability model differs from a validation for a presence/absence model. Validating a suitability is about presence in suitable areas with no record of presence in non suitable areas. The absence of a record in a suitable areas does not mean that the area is not suitable. Validation of the malaria Transmission Suitability Index was restricted to areas where entomological studies had previously occurred, with the results also verified by entomological experts.

Expert validation consisted of the following: direct comparison with previously collected presence data and expert opinion on the suitability of the model structure and scores. Over the period 1968 to 1973, Brian Taylor and Mario Maffi surveyed mosquito presence over much of the Solomon Islands [6]. Although locations were provided for each mosquito species encountered, the data were not in a format that allowed it to be converted into GIS locations.

Dr. Nigel Beebe and his collaborators at the University of Queensland provided data with which to valid the model. His team sampled water bodies along the coast and inland in north Guadalcanal at the end of the dry season (October to November) 1997 [15]. The provided data consisted of GPS coordinates, including elevation, of all samples linked to the presence or absence of *An. farauti*. Model validation using this data indicated that there was no significant difference between Beebe presence data and that produced by the TSI model (81% of the presence points described by Beebe et al. had TSI scores > 9, while only 16% of the absence points had TSI scores > 9). As the data by Beebe et al. [15] was based on sampling only sites along roadsides on a single night, ‘absence’ points do not conclusively mean the mosquito was not present at that location.

The study by Russell et al. [9], which sampled larvae in Central Province and Western Province in Solomon Islands from December 2011 to December 2012 was better adapted to validation of the TSI model, as it reduced the false-absence bias by repeated monthly sampling over 10 consecutive days in five sites in the north side of a lagoon and also monitored larval density [26].

**Fig. 6:**
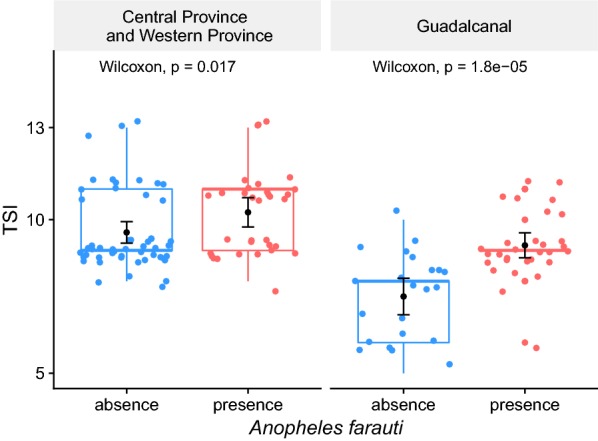
*Anopheles farauti* presence and absence comparison TSI means test for Central Province and Western Province, from Russell et al. [9], and for Guadalcanal, from Beebe et al. [15]. Mean and confidence intervals (95%) are in black, boxplot are in colour, dots are representing TSI values for each sample, note that these values have been jittered (random noise added around the integer values to avoid overplotting)

For both studies by Beebe et al. and Russell et al., locations where *An. farauti* larvae were collected had a significantly higher TSI score than for locations where collections than where no *An. farauti* can be found (Fig. 6 and Table 2).

**Table 2:** *Anopheles farauti* presence and absence comparison TSI means test

*Anopheles farauti*	Presence (TSI mean (*n*))	Absence (TSI mean (*n*))	Wilcoxon test
Guadalcanal	9.17 (*31*)	7.50 (*21*)	p < 0.0001
CP and WP	10.24 (*33*)	9.59 (*51*)	p < 0.02

Based on a total of 52 collections sites in Guadalcanal Province from Beebe et al. [15] for the year 1997 and of a total of 84 collection sites in Central Province and Western Province (CP and WP) from Russell et al. [9]

The TSI mean in Central Province and Western Province is higher than in Guadalcanal (p < 0.0001), most likely because villages in Central and Western Province are within 2 km from the coast and, therefore, so are the sampled sites. In Beebe et al. [15], positive sites have been found up to 6.4 km from the coast.

Validation by expert consultation of the suitability of the TSI model and scores selected was conducted with Professor Tom Burkot (Australian Institute of Tropical Health and Medicine, James Cook University) and Lieutenant Colonel Robert Cooper (Australian Army Malaria Institute).

When additional data on the presence/absence of *An. farauti* becomes available it is hoped that further model validation will be possible, including for additional regions.

## Discussion

Malaria remains a serious health risk to many communities within the Solomon Islands. With environmental factors accounting for around 90% of the malaria transmission risk, and approximately two-fifths of the global malaria burden being attributed to environment factors that are modifiable [27], there is an opportunity to provide tools to help reduce this risk. Tools, including GIS, can greatly assist in the monitoring, surveillance, and prediction of malaria presence [28]. The TSI developed here concentrates on the development of a model focused on the environmental component of malaria transmission risk.

Previous malaria surveillance models have been developed, including for the Pacific region (e.g. [5, 29]). The GIS-based spatial decision support system of Kelly et al. was applied to three Pacific provinces including two for the Solomon Islands, Temotu and Isabel. In their system, Kelly et al. [5] mapped confirmed malaria cases and used this to classify active transmission foci for use in guiding targeted responses in eliminations zones. Rosewell et al. [29] also developed a case-based malaria surveillance system based on mobile technologies and GIS for Papua New Guinea. Statistical algorithms were used to generate outbreak detection data down to the village level, with the results being automatically displayed within a mapping platform. In the Solomon Islands, not all cases of malaria are currently reported within the central government’s Health Information System, reasons including illegible writing, lack of understanding of the clinical definition of malaria and insufficient resources and training [30] and this can impact on the usefulness of malaria surveillance models such as those discussed here. In addition, both systems of Kelly et al. and Rosewell et al. [5, 29] support the rapid detection of malaria cases but, unlike the TSI developed here, neither has any predictive capacity or considers the environmental preferences of the mosquitos and malaria parasite transmission. This includes the reported cases of malaria.

Community involvement is a critical component in malaria prevention and control [28], with eradication on small isolated islands being possible when levels of community involvement are high [31]. Community involvement can also extend to treatment support, including early recognition of fever, active case detection and treatment distribution support. This self-monitoring can provide useful data for GIS-based tools with the tools providing support to the communities engaged in malaria control efforts [28].

The malaria TSI developed here is a useful tool for the detection of locations at high risk of malaria transmission. Unlike previously available information on malaria risk for the Solomon Islands, e.g. that based on the reported number of cases for health districts, the TSI is provided at a much higher spatial resolution. This can enable malaria control measures to be more effectively targeted, especially vector control, down to the scale of settlements, with the potential to greatly reduce resource expenditure and use.

Although the malaria TSI model performed well on the data available, the validation with the presence data of previous published studies was restricted to only some parts of the Solomon Islands and with sampling methods not always compatible with the study. Species detectability can vary in space and time and absence records depends on the sampling methods used [18, 32], suitability information is not the same as presence prediction and does not represent a probability of occurrence. *Anopheles farauti* is known to prefer established breeding sites [33] and has a narrow and restrictive distribution [12], with the sites being fixed [9], allowing a great variation of the average density between villages [34]. Environment can also play a role in isolation of breeding sites. In their first oviposition cycle, *An. farauti* females tend to disperse less to locate their hosts or breeding sites when released in their natal village of capture than when released in another village [11]. This demonstrates that *An. farauti* has the potential to widen its distribution but that they prefer to stay where they are, if the environment is suitable and the hosts are present. As with any model, the model development and validation is only as good as the data input and could be improved with new information, such as field or experimental entomological or environmental data or with new tools.

It is currently not possible to effectively incorporate malaria incidence data into a model coupled with the TSI, as no information is currently available that breaks down the health district records, compiled over a wider catchment area, to the spatial and environmental resolution of the TSI. As additional data on malaria cases, and the presence and absence of the mosquito vector, become available it will be possible to further refine and validate the TSI, including the potential to incorporate the TSI into existing surveillance systems such as that developed by Kelly et al. [5] or Rosewell et al. [29]. The next step would be to add EIR quantitative adult as possible and malaria incidence cases information layers.

To some, the malaria TSI model developed here may appear simple. However, this also provides an advantage as the model is structured in such a way that it is easy to modify existing parameters, and their weights, or add additional parameters as new information comes to hand through either updated census, elevation or land cover maps or in the form of new information on the mosquito or its parasites environmental preferences. These are currently not explicitly included in the model as hydrometeorological factors provide a better means of studying temporal variability of mosquito habitats. However, variations in climate variables, such as rainfall, have been known to influence the risk of malaria, including in the Solomon Islands [26, 35], and may offer some cost efficiencies to malaria control programmes through early warning systems [35, 36].

## Conclusion

The TSI model developed here provides assistance for those wishing to make predictions of likely incidence of malaria outbreaks based on the environmental preferences of the mosquito, *An. farauti*. These predictions can provide sufficient lead-time for agencies to target malaria prevention and control measures and can assist with effective deployment of often limited resources.

Based on literature and interviews with experts, three parameters were identified as influencing the likelihood of malaria mosquito vector presence. These were: distance from coastline, elevation and presence of water bodies. The model was developed for the Solomon Islands and ecological preferences of *An. farauti,* but can be modified for other regions and species. The final TSI had a spatial resolution of 250 m, providing a much greater spatial resolution than any previous predictive models for the Solomon Islands, allowing for the scale of the control programme to move from districts to villages. This will allow those tasked with malaria control and elimination to identify spatial clusters of likely outbreaks and to define strategies to potentially eliminate malaria at the periphery of these and to reduce transmission of malaria between clusters.

In order for the Transmission Suitability Index to remain current, it is recommended that it be updated as new information on the environmental preferences of *An. farauti* becomes available, for example, after each georeferenced census period. In the future it is hoped that the TSI developed here can be incorporated into other malaria surveillance tools, so that locations with predicted malaria outbreaks can be superimposed with updated case data and information at high resolution on past control measures, such as spraying and control effectiveness.

In summary, the TSI, provides a visual display of likely malaria outbreaks allowing quick and effective communication of malaria risk and is a useful tool for assisting decision-makers with both a national coverage and at the village scale.

## Figures and Tables

**Fig. 7:**
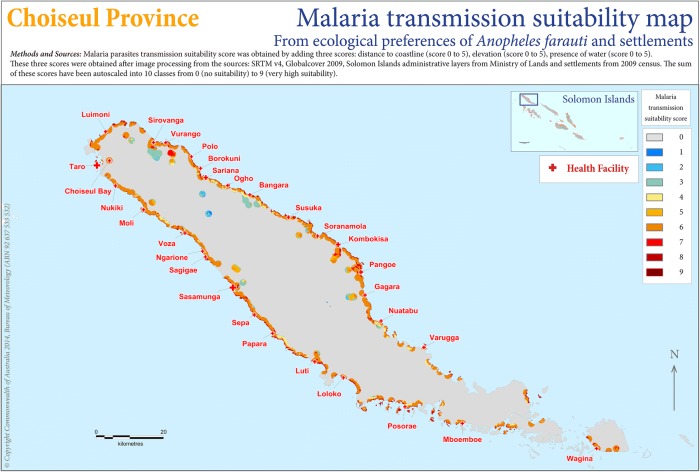
Choiseul Province

**Fig. 8:**
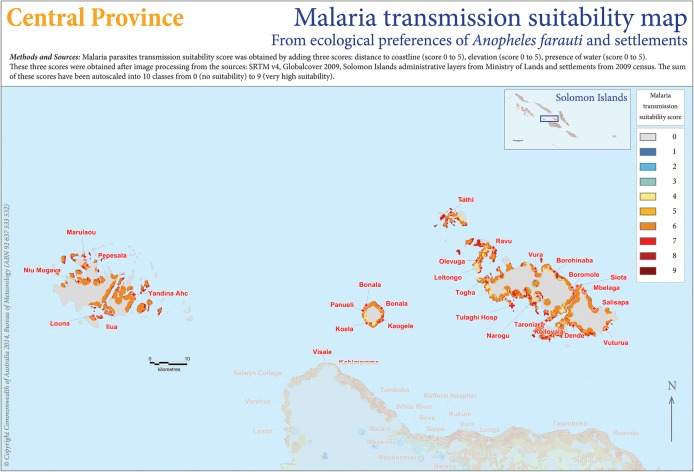
Central Province

**Fig. 9:**
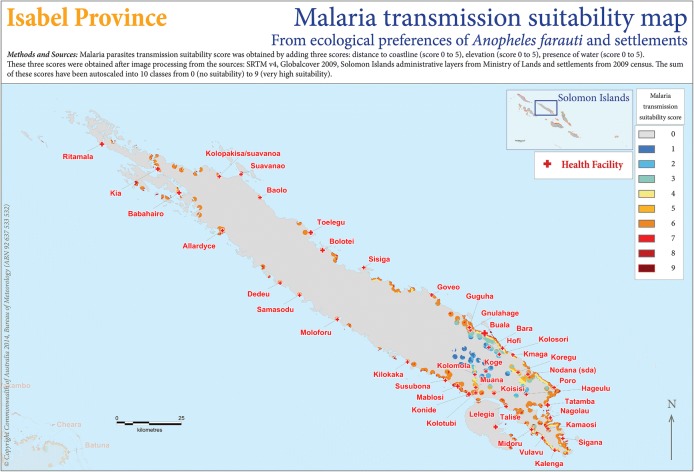
Isabel Province

**Fig. 10:**
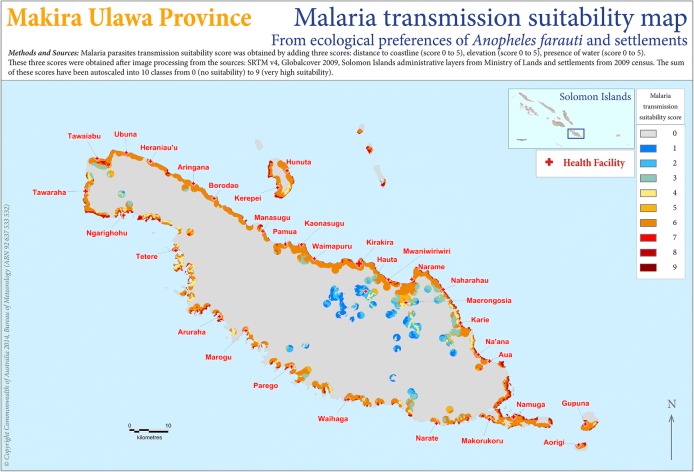
Makira Ulawa Province

**Fig. 11:**
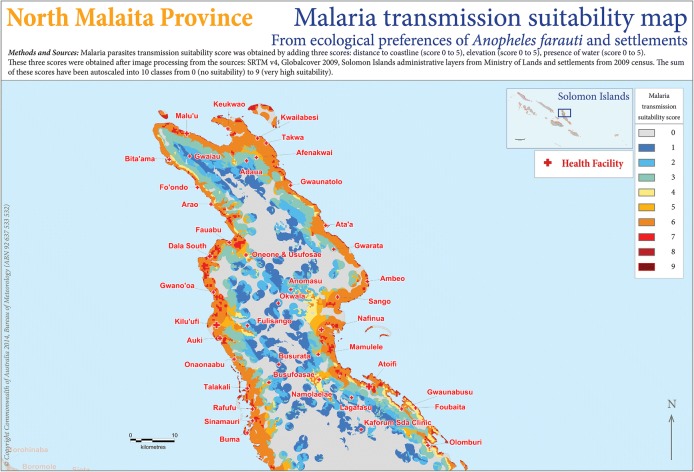
North Malaita Province

**Fig. 12:**
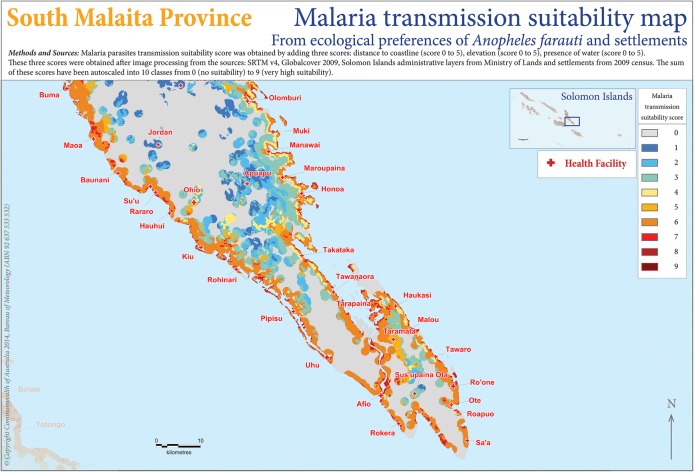
South Malaita Province

**Fig. 13:**
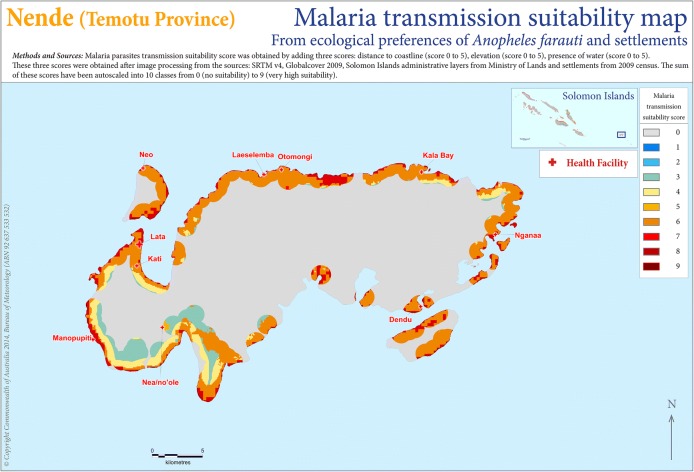
Nende, main island of Temotu Province

**Fig. 14:**
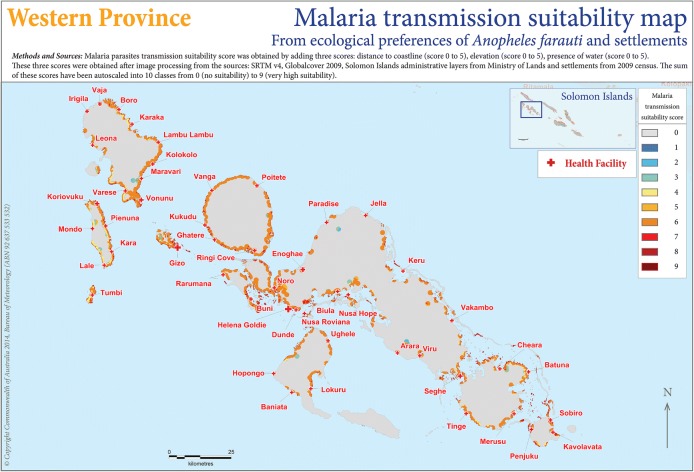
Western Province

## Appendix A

- Additional details of images and datasets used in the analyses.
- LANDSAT ETM + band 8:

- NASA Landsat Program, 2003, Landsat ETM + scene p087r067_7p20010404_z58_nn80, SLC-Off, USGS, Sioux Falls, 04/04/2001.
- NASA Landsat Program, 2003, Landsat ETM + scene p088r067_7p20010404_z58_nn80, SLC-Off, USGS, Sioux Falls, 04/04/2001.
- NASA Landsat Program, 2003, Landsat ETM + scene p087r066_7p20010404_z58_nn80, SLC-Off, USGS, Sioux Falls, 04/04/2001.
- NASA Landsat Program, 2003, Landsat ETM + scene p088r066_7p20010404_z58_nn80, SLC-Off, USGS, Sioux Falls, 04/04/2001.
- NASA Landsat Program, 2003, Landsat ETM + scene p083r067_7p20070307_z58_nn80, SLC-Off, USGS, Sioux Falls, 07/03/2007.
- NASA Landsat Program, 2003, Landsat ETM + scene p083r068_7p20070307_z58_nn80, SLC-Off, USGS, Sioux Falls, 07/03/2007.

- Elevation data

- USGS, year, Shuttle Radar Topography Mission, 3 arcs second, version 4, srtm_68_14, Global Land Cover Facility, University of Maryland, College Park, Maryland, Month year.
- USGS, year, Shuttle Radar Topography Mission, 3 arcs second, version 4, srtm_69_14, Global Land Cover Facility, University of Maryland, College Park, Maryland, Month year.
- USGS, year, Shuttle Radar Topography Mission, 3 arcs second, version 4, srtm_69_15, Global Land Cover Facility, University of Maryland, College Park, Maryland, Month year.
- USGS, year, Shuttle Radar Topography Mission, 3 arcs second, version 4, srtm_70_15, Global Land Cover Facility, University of Maryland, College Park, Maryland, Month year.

- Landcover data

- Globcover14_200901_200912_V2.
- © ESA 2010 and UCLouvain, Accompanied by a link to our ESA DUE GlobCover website: http://due.esrin.esa.int/page_globcover.php.

The Internet Archive

The Internet Archive is a 501(c) (3) non-profit library.

https://archive.org/search.php?query=anopheles%20farauti.

Walter Reed Institute

The Walter Reed Biosystematics Unit| Museum Support Center, MRC-534 | Smithsonian Institution| 4210 Silver Hill Rd.| Suitland, MD 20746-2863 USA| Ph: 301-238-1077; FAX: 301-238-3168 Entomology Branch| Walter Reed Army Institute of Research| 503 Robert Grant Avenue| Silver Spring, MD 20910-7500 USA.

http://www.wrbu.org/mqID/mq_medspc/AD/ANfar_hab.html.

## Appendix B

Malaria transmission suitability maps. See Figs. 7, 8, 9, 10, 11, 12, 13 and 14.

## Abbreviations

DDT: dichlorodiphenyltrichloroethane
ETM: enhanced thematic mapper
GADM: database of global administrative areas
GIS: geographical information system
GPS: global positioning system
NVBDCP: National Vector Borne Disease Control Programme
SRTM: Shuttle Radar Topography Mission
SLC: scan line corrector
TSI: transmission suitability index
