# Correction to: Mapping a Plasmodium transmission spatial suitability index in Solomon Islands: a malaria monitoring and control tool

**DOI:** 10.1186/s12936-018-2580-2

**Published:** 2018-11-19

**Authors:** Isabelle Jeanne, Lynda E. Chambers, Adna Kazazic, Tanya L. Russell, Albino Bobogare, Hugo Bugoro, Francis Otto, George Fafale, Amanda Amjadali

**Affiliations:** 1 Australian Bureau of Meteorology, Melbourne, VIC Australia; 2 Australian Institute of Tropical Health and Medicine, James Cook University, Cairns, QLD Australia; 3 Ministry of Health and Medical Services, National Vector Borne Disease Control Programme, Honiara, Solomon Islands; 4 Research Department, Solomon Islands National University, Honiara, Solomon Islands; 5 Pacific Science Solutions, Suva, Fiji

## Correction to: Malar J (2018) 17:381 10.1186/s12936-018-2521-0

Following publication of the original article [1], one of the authors flagged that the images for Figs. 2 and 3 were swapped in the published article—Fig. 2 had the image meant for Fig. 3 and vice versa.

As such, the original article [1] has been updated to correct this error.

The publisher apologizes for any inconvenience caused.
